# Sustainable Additive Manufacturing: Mechanical Response of Polyamide 12 over Multiple Recycling Processes

**DOI:** 10.3390/ma14020466

**Published:** 2021-01-19

**Authors:** Nectarios Vidakis, Markos Petousis, Lazaros Tzounis, Athena Maniadi, Emmanouil Velidakis, Nikolaos Mountakis, John D. Kechagias

**Affiliations:** 1 Mechanical Engineering Department, Hellenic Mediterranean University, 71410 Heraklion, Crete, Greece; vidakis@hmu.gr (N.V.); mvelidakis@hmu.gr (E.V.); mh90@edu.hmu.gr (N.M.); 2 Department of Materials Science and Engineering, University of Ioannina, 45110 Ioannina, Greece; latzounis@uoi.gr; 3 Department of Materials Science and Technology, University of Crete, 70013 Heraklion, Crete, Greece; maniadi@materials.uoc.gr; 4 General Department, University of Thessaly, 41500 Larissa, Greece; jkechag@uth.gr

**Keywords:** additive manufacturing (AM), three-dimensional (3D) printing, recycling, polyamide 12 (PA12), tensile test, flexural test, Charpy’s impact test, Vicker’s micro-hardness, scanning electron microscopy (SEM)

## Abstract

Plastic waste reduction and recycling through circular use has been critical nowadays, since there is an increasing demand for the production of plastic components based on different polymeric matrices in various applications. The most commonly used recycling procedure, especially for thermoplastic materials, is based on thermomechanical process protocols that could significantly alter the polymers’ macromolecular structure and physicochemical properties. The study at hand focuses on recycling of polyamide 12 (PA12) filament, through extrusion melting over multiple recycling courses, giving insight for its effect on the mechanical and thermal properties of Fused Filament Fabrication (FFF) manufactured specimens throughout the recycling courses. Three-dimensional (3D) FFF printed specimens were produced from virgin as well as recycled PA12 filament, while they have been experimentally tested further for their tensile, flexural, impact and micro-hardness mechanical properties. A thorough thermal and morphological analysis was also performed on all the 3D printed samples. The results of this study demonstrate that PA12 can be successfully recycled for a certain number of courses and could be utilized in 3D printing, while exhibiting improved mechanical properties when compared to virgin material for a certain number of recycling repetitions. From this work, it can be deduced that PA12 can be a viable option for circular use and 3D printing, offering an overall positive impact on recycling, while realizing 3D printed components using recycled filaments with enhanced mechanical and thermal stability.

## 1. Introduction

Polymers are materials widely used by various industries nowadays, due to their relatively low-cost as well as unique intrinsic properties, such as ease of processability and light weight character, resulting into products with high mechanical, electrical and thermal properties [1,2,3]. As the demand for plastic products grows rapidly, the global manufacturing of plastics is increasing at an approximately annual rate of 8.2% [4]. To date, more than 3 billion tons of plastics has been produced globally [5], and the increasing use of plastic products leads to a larger quantity of plastics ending up as waste in the environment. Several strategies exist on how waste plastic should be used, such as recycling, reuse and resource and energy recovery [6]. Amongst all, the one that is considered to be the most cost efficient and effective and deemed to be consistent enough for being implemented by the plastics’ industry is the mechanical recycling [7,8,9].

Fused Filament Fabrication (FFF) is the most commonly used 3D printing AM method to date, especially for thermoplastic in nature polymeric materials, mainly due to characteristics, such as rapid processing, simplicity and cost-efficiency [10,11]. Research is available but limited regarding the recycling of thermoplastics by repetitive melt extrusion cycles and consecutive 3D FFF printing, while basically studies exist only for the most commonly used thermoplastics in FFF such as acrylonitrile butadiene styrene (ABS) [12] and polypropylene (PP) [13].

Typical materials as filaments used in Fused Filament Fabrication nowadays are engineering thermoplastic polymers, such as polylactic acid (PLA) [14] and acrylonitrile butadiene styrene (ABS) [15,16,17,18,19,20], with extensive literature available for their mechanical, electrical and thermal properties. Literature is not yet enriched enough with data on the mechanical and thermal properties of semi-crystalline materials such as polyamide 6/66/12 (PA 6/66/12) used for 3D printing via FFF, with only a few reports available on the tensile strength and impact strength [21,22,23] as well as on the flexural strength [24]. This is mainly due to the difficulties of the material’s processing in FFF 3D printing, i.e., (i) distortion and warping of these materials, and (ii) upon their crystallization, they tend to have volume reduction associated with the formation of ordered, more densely packed regions during crystallization, when compared to amorphous polymers, such as ABS [25,26,27]. Consequently, crystallization, as a mechanism, is believed to drastically decrease molecular chain mobility and thus can prevent interlayer fusion to establish sufficiently strong adhesion between the 3D printed layers.

Regarding continuous recycling, research exists for the mechanical and thermal properties of recycled ABS [12], while literature in not yet available on the continuous recycling of semi-crystalline thermoplastic polymeric materials such as polyamides for 3D printing applications. Moreover, the occurring mechanisms upon recycling based on melt processing methods, i.e., change of crystallinity, scission of polymeric macromolecular chains, etc., which can significantly affect the mechanical properties of the 3D printed recycled materials and their thermal stability have not been yet fully elaborated.

Polyamide (PA) contains the amide repeat sequence linkage in the polymeric backbone, as well as H-bonds that are formed between the adjacent polymeric chains. Different types of polyamides exist, based on the macromolecular chains’ architectures and the main chemistry of the backbone, e.g., PA66, PA6,12, PA12, etc. As such, polyamides are typically tough, semi-crystalline polymers, with low glass transition temperature (T_g_). Moreover, polyamides are known to be extensively used in automotive industry to manufacture automobile parts, as well as in textile industry and fabrics, due to their superior specific mechanical properties such as impact strength and their ease of processability [28].

**Figure 1:**
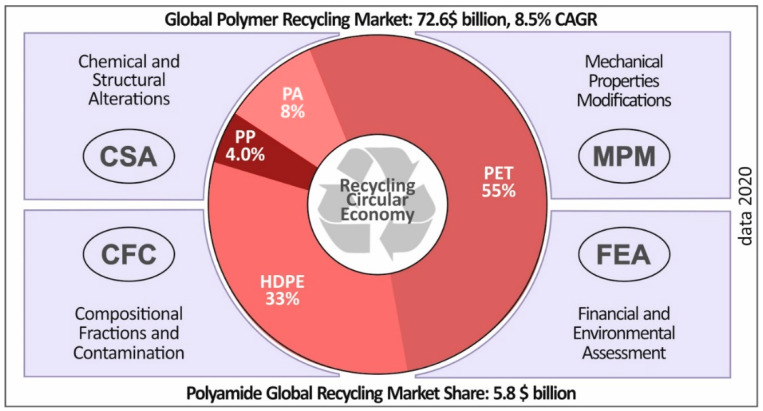
2020 recycled plastics market and parameters affecting plastics circular economy (market volume data source: Technavio. Global Recycled Plastics Market 2020–2022 (IRTNTR21968).

Literature is available on the recycling of different types of polyamides and polyamide-based composites consisting of recycled polyamide [29,30,31,32,33,34]. However, no research is available on the recycling of virgin PA12 for a number of recycling courses and its effect on the mechanical and thermal properties of 3D printed specimens. Furthermore, from an economical point of view, besides the environmental necessity to recycle this type of polymer, polyamide (polyamide group of Plastics) holds the 8% of the global recycling market share, translating to 5.8 billion dollars in 2020 (Figure 1), creating thus a strong interest for recycling.

The current research work seeks initially to promote the production of recycled, sustainable PA12 filaments for circular use and to identify the underlying mechanisms that directly affect the mechanical and thermal stability of the recycled polymers. Moreover, this study aims to enrich literature with data upon the mechanical and thermal properties of 3D printed PA12 over multiple recycling courses, proving that the continuous recycling process for a certain number of repetitions can increase the mechanical properties of the recycled polyamide, when compared to virgin material. This research work focuses on the behavior of virgin and recycled PA12 material throughout six (6) recycling courses, with specimens manufactured via FFF, 3D printing, to investigate the thermomechanical process impact on the mechanical strength of the produced specimens as well as the structural and morphological changes, resulting from the alteration of crystallinity and thermal properties. Mechanical testing (tension, flexion, impact, micro-hardness) was employed to evaluate the effects of degradation processes in the mechanical properties of the specimens made with virgin and recycled filament.

It was found that there is an overall increase in the mechanical properties throughout the recycling courses until the 5th recycling course, both in tension and in flexion. For the corresponding thermal properties, thermogravimetric analysis (TGA) and differential scanning calorimetry (DSC) were performed, while Scanning Electron Microscopy (SEM) was conducted for the evaluation of their morphological characteristics in all recycling courses studied. DSC analysis showed a slight decrease in the material’s crystallinity after the 4th recycling course. Morphological analysis indicated that after the 4th recycling course, 3D printed specimens had degraded interlayering fusion, cause of the minor inability of the material to flow due to possible increased of crystals’ size affecting the melt rheological properties of the polymer. With the data derived from this study, it was shown that there is an economical and a manufacturing benefit in recycling at least up to five times of PA12 and especially a commercially available PA12 material used for FFF Additive Manufacturing.

## 2. Materials and Methods

### 2.1. Materials

A polyamide from Arkema (Colombes, France) was used and particularly PA12 resin AESNO TL grade. Arkema’s PA12 is sold under Rilsamid PA12 AESNO TL brand name. The fundamental properties of this PA12, as they are shown in the vendor’s corresponding Technical Sheet, include a density of 1.01 g/cm^2^ (according to ISO 1183), Melt Volume-Flow Rate (MVR) of 8.0 cm^3^/10 min (according to ISO 1133) at 235 °C/5.0 kg, Vicat Softening Temperature at 142 °C (according to ISO 306/B50) and Melting Temperature at 180 °C (according to ISO 11357-3). According to the vendor, in this PA12 grade, some additives have been added as for heat stabilizing, lubrication, and UV stabilization.

### 2.2. Methods

#### 2.2.1. Recycling Process

**Figure 2:**
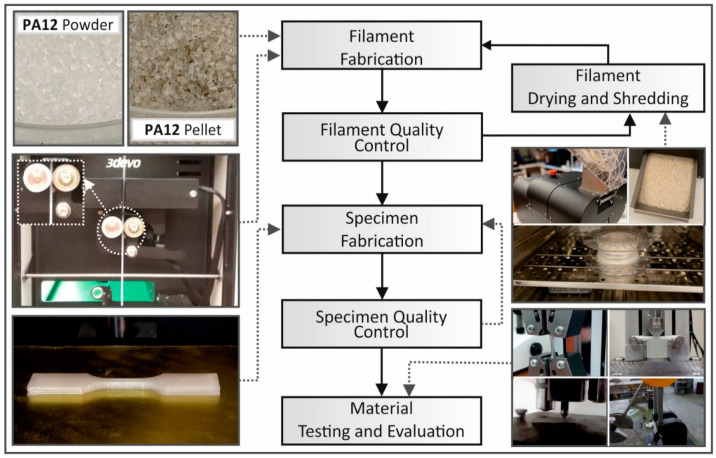
Flow chart with the methodology followed for the PA12 recycling in the current study.

In this study, a procedure of repeated extrusion processes was followed in order to simulate the degradation mechanism of consequent recycling process [35]. The material selected for this work, PA12, was purchased from Arkema (Colombes, France), in the form of fine granules. After a drying procedure at 80 °C for 24 h, granules were extruded through a single screw extruder (3D Evo Composer 450, 3D Evo B.V., Utrecht, The Netherlands), in order to produce 1.75 mm diameter filament appropriate for FFF 3D printing. The produced filament was then dried at the same conditions, and part of it was used to produce 3D printed specimens, while the rest of the filament was shredded back into pellets in order to be recycled. The above procedural steps were repeated 6 times, otherwise defined, and denoted in this work as 6 corresponding recycling courses. In each recycle course, filament quality control was performed under a real-time monitoring system working with optical sensors (extruder’s built-in system). A deviation of 0.07 mm on average was measured in each recycle course’s produced filament. Quality control was also conducted under optical and dimensional measures in each manufactured specimen. Figure 2 summarizes all processes followed for the purposes of this study.

The shredder machine that was utilized in order to reshape the filament into pellets was a Shreddit (3D Evo B.V., Utrecht, The Netherlands). Through all recycling courses, the extrusion parameters in the 3D Evo Composer 450 (3D Evo B.V., Utrecht, The Netherlands) were kept constant. More specifically, heat zone 1 (closer to extruder’s nozzle) was set to 210 °C, heat zone 2 was set to 220 °C, heat zone 3 was set to 220 °C and finally heat zone 4 (closer to extruder’s hopper) was set to 185 °C. Extruder’s screw rotational speed was set to 8.5 rpm, while the cooling fans after the nozzle were set to 50% speed.

**Figure 3:**
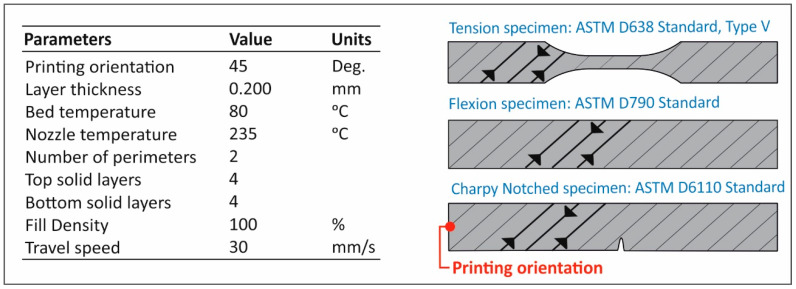
Parameters employed in the study for the construction of the FFF 3D printed specimens.

In this study, Fused Filament Fabrication was selected to manufacture specimens for each recycling course. Specimens were fabricated using a Craftbot Plus (Craftbot ltd, Budapest, Hungary) Fused Filament Fabrication technology 3D Printer, with the all-metal 3D printing head (hot end) in its setup. The 3D printing parameters are summarized in Figure 3. It has to be mentioned that a masking tape (3M 101+) was used over the aluminum build-plate (heatbed) in order to reduce the wrapping effect on the PA12. Finally, no cooling (3D printer nozzle’s fans were set to 0%) was used.

#### 2.2.2. Tensile Specimens’ Fabrication and Testing

**Figure 4:**
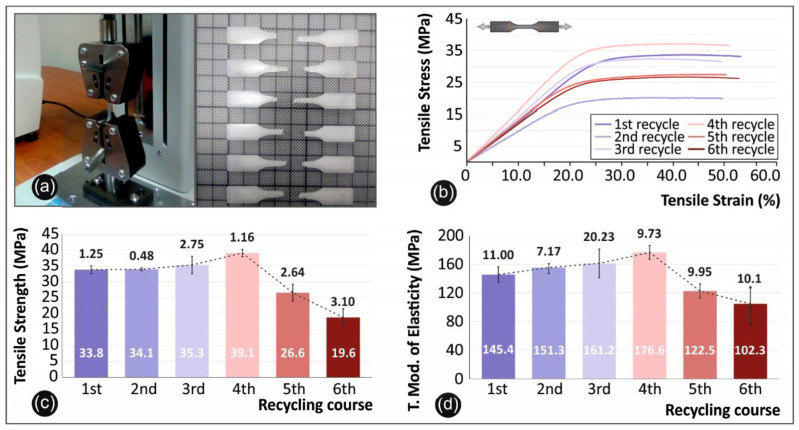
Tensile tests: (**a**) device experimental setup and specimens after the test, (**b**) stress strain graphs from the no 2 specimen of each recycle course, (**c**) comparison of the tensile strength average values along with the calculated deviation from each recycle course studied, and (**d**) comparison of the tensile modulus of elasticity average values along with the calculated deviation from each recycle course studied.

Tensile experiments were carried out under the ASTM D638-02a international standard. According to the standard, a type V specimen of 3.2 mm thickness was chosen, and a total of six (6) specimens were manufactured and tested for each recycling course. Tensile test’s device used for this purpose was an Imada MX2 (Imada inc., Northbrook, IL, USA) tension/flexion test apparatus in tensile mode set up, using standardized grips (Figure 4a). Elongation speed for the tensile test was set to 10 mm/min as per the standard specifications and the experimental lab temperature when tests were conducted was 21 °C.

#### 2.2.3. Flexion Specimens’ Fabrication and Testing

**Figure 5:**
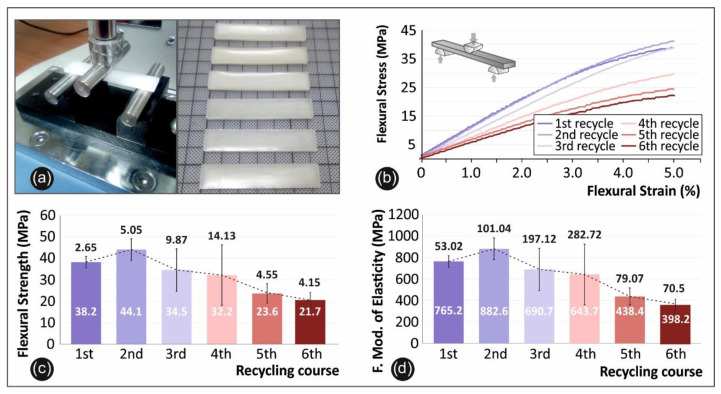
Flexural tests: (**a**) device experimental setup and specimens after the test, (**b**) stress strain graphs from the no 2 specimen of each recycle course, (**c**) comparison of the flexural strength average values along with the calculated deviation from each recycle course studied, and (**d**) comparison of the flexural modulus of elasticity average values along with the calculated deviation from each recycle course studied.

Flexural tests were conducted according to the ASTM D790-10 international standard. A total of six (6) specimens for each recycling course were fabricated with a thickness of 3.2 mm and tested in the same device referred above in three-point bending test setup. Imada’s chuck speed was set to 10 mm/min, and the flexural setup used is presented in Figure 5a. The experimental lab temperature when tests were conducted was 21 °C.

#### 2.2.4. Impact Specimens’ Fabrication and Testing

Impact tests were conducted according to the ASTM D6110-04 international standard. Specimens were built with dimensions of 80 mm length, 8 mm width and 10 mm thickness. A total of six (6) specimens for each recycling course were tested in a Terco MT 220 (Terco AB, Huddinge, Sweden) Charpy’s impact apparatus. Release height of the apparatus hammer was the same for all the experiments. The experimental lab temperature when tests were conducted was 21 °C.

#### 2.2.5. Micro-Hardness Measurements

Microhardness measurements were conducted according to the ASTM E384-17 international standard. The specimens’ surface was fully polished before each set of measurements according to the standard’s requirements. An Innova Test 300 Vickers (Innovatest Europe BV, Maastricht, The Netherlands) was used. Applied force was set to 100 gF and a duration of 10 s was selected for indentation. Imprints were measured under six (6) different specimens for each one of the six (6) recycle courses.

#### 2.2.6. Thermal Analysis

Thermogravimetric analysis (TGA) under oxygen was performed to obtain information about the critical degradation temperature of the PA12 virgin material selected for this research to identify the appropriate extrusion and 3D printing temperature. The measurements were carried out with a Perkin Elmer Diamond TG/TDA (PerkinElmer, Inc., Waltham, Massachusetts, United States of America) with a heating cycle from 32 °C to 550 °C with a heating step of 10 °C/min.

Differential scanning calorimetry (DSC) was also performed to obtain information about the effect of recycling, on the melting point (T_m_) and the shift in the degree of crystallinity of the samples. The measurements were taken via a Perkin Elmer Diamond DSC (PerkinElmer, Inc., Waltham, Massachusetts, United States of America) with a temperature cycle of 50 °C to 300 °C with a heating step of 10 °C/min and then cooling down to 50 °C.

#### 2.2.7. Morphological Characterization

Scanning electron microscopy (SEM) characterization was performed for the morphological characterization of specimens’ internal/external structure and interlayer fusion via fractured surfaces’ microstructural analysis and 3D printed samples side surface morphology, respectively. The SEM analysis was carried out using a JEOL JSM 6362LV (Jeol Ltd., Peabody, MA, USA) electron microscope in high-vacuum mode at an accelerating voltage of 5 kV.

## 3. Results

### 3.1. Tension Results

In Figure 4 below, the tensile testing setup utilized in this work is depicted (Figure 4a), alongside with the fractured specimens after the tensile test experiments (Figure 4a), the comparative graphs of the tensile stress versus strain (Figure 4b), the average tensile strength and the deviation in each recycling step (Figure 4c) and the average tensile modulus of elasticity and deviation (Figure 4d) for all six (6) recycling courses.

### 3.2. Flexion Results

In Figure 5 below, the flexural testing setup utilized in this work is presented (Figure 5a), alongside with the specimens after the experiment (Figure 5a), the comparative graphs of the flexural stress versus strain (Figure 5b), the average flexural strength and the deviation in each recycling step (Figure 5c) and the average flexural modulus of elasticity and deviation (Figure 5d) for all six (6) recycling courses.

### 3.3. Impact Results

**Figure 6:**
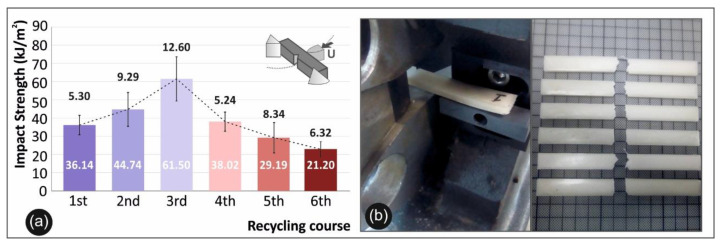
Impact tests: (**a**) comparison of the impact strength average values along with the calculated deviation from each recycle course studied and (**b**) device experimental setup and specimens after the test.

Charpy’s notched Impact Test results are shown in Figure 6a. Specifically, in Figure 6a, the mean calculated impact strength of each recycling course for PA12 is presented, while Figure 6b presents the Charpy’s test setup and broken specimens after the experiment.

Recycling courses comparative graphs (a) calculated average values and deviation of the impact strength for all the recycling courses studied and (b) Charpy’s impact test experimental setup alongside with the broken specimens.

### 3.4. Micro-Hardness Results

**Figure 7:**
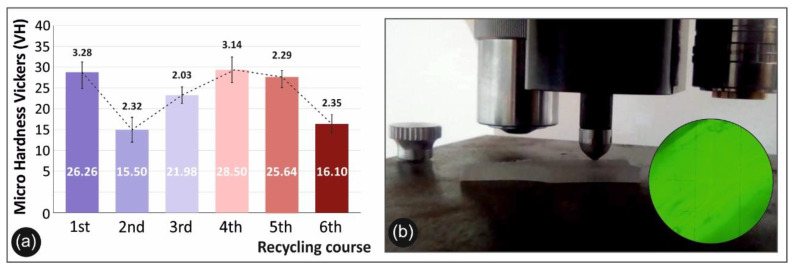
Micro-hardness Vickers tests: (**a**) comparison of the Vickers micro-hardness average values along with the calculated deviation from each recycle course studied and (**b**) device experimental setup and specimens after the test.

Figure 7a below presents the mean micro-hardness Vickers (HV) values calculated for each recycling course of PA 12, while the experimental setup is depicted in Figure 7b.

### 3.5. Thermal Analysis Results

**Figure 8:**
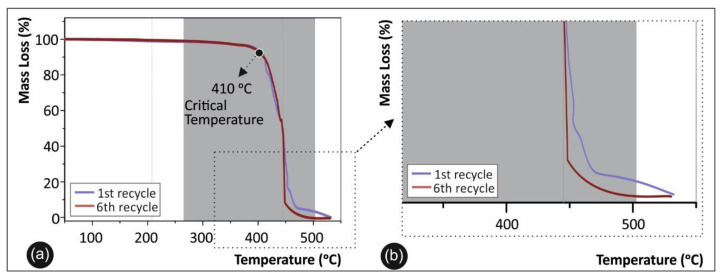
(**a**) TGA graphs for pure PA12 1st recycling course versus 6th recycling course; (**b**) magnified TGA temperature window for the material’s thermal degradation region.

Regarding the thermal analysis, the TGA results of pure PA12 in direct comparison with PA12 of the 6th recycling course are presented in Figure 8a, while a zoom in the degradation area is depicted in Figure 8b.

**Figure 9:**
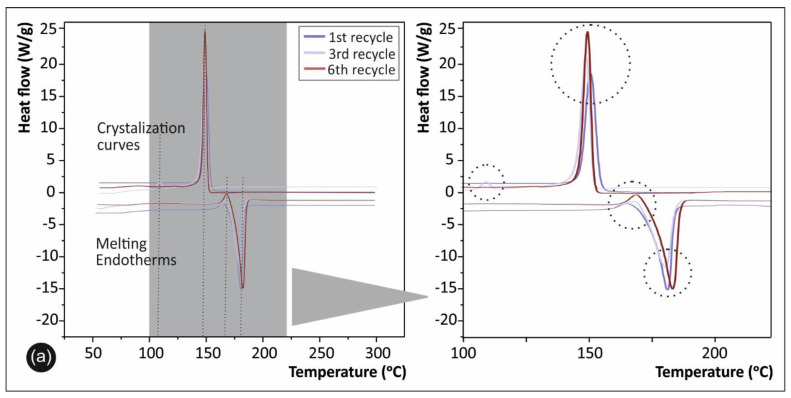
(**a**) DSC 2nd heat/cool cycle curves for PA12 1st, 3rd, and 6th recycling courses and (**b**) magnified temperature window of the DSC curves.

DSC analysis was employed for the qualitative determination of the degree of crystallinity, as well as the melting temperature (T_m_) and glass transition temperature (T_g_) of the investigated materials. The degree of crystallization was calculated by the following equation [36]:

(1) $$X_{c}\left(\%\mathrm{crystallinity}\right)=\frac{\Delta H_{m}}{\Delta H_{o}}*100\%$$

whereas ΔH_m_ is the melting enthalpy (the area under the melting curve), and ΔH_o_ is a theoretical value of the melting enthalpy of 100% crystalline PA12. The value ΔH_o_ = 230 J/g was used in a degree of crystallinity calculation. DSC results are shown in Figure 9a and Table 1, while a magnification of a specific temperature window of the graph is depicted in Figure 9b.

### 3.6. Morphological Characterization Results

**Figure 10:**
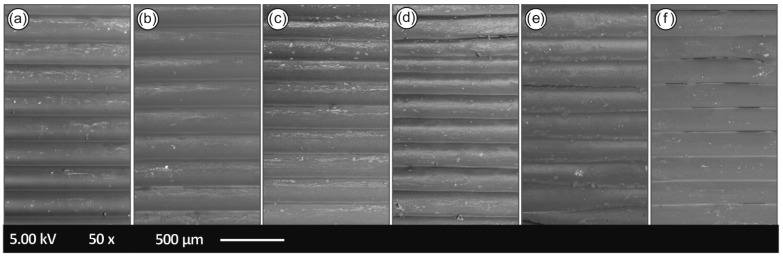
Specimens’ side surface SEM images: (**a**) 1st recycling course, (**b**) 2nd recycling course, (**c**) 3rd recycling course, (**d**) 4th recycling course, (**e**) 5th recycling course and (**f**) 6th recycling course.

In the following Figure 10, the SEM images of the tensile specimens’ side surface is depicted in order to identify any defects in the layering of the specimens as well as to examine the layering interfusion of the specimens after each recycling course.

**Figure 11:**
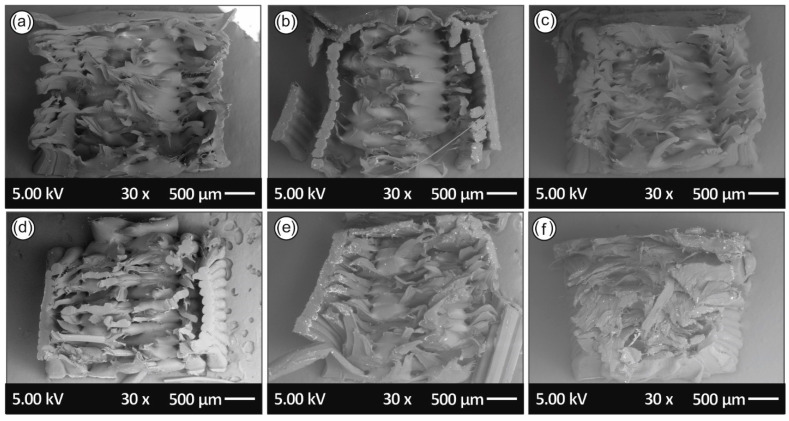
Tensile specimens’ fracture surface SEM images: (**a**) 1st recycling course, (**b**) 2nd recycling course, (**c**) 3rd recycling course, (**d**) 4th recycling course, (**e**) 5th recycling course and (**f**) 6th recycling course.

In Figure 11, the fractured areas of the tensile specimens, one of each recycling course are depicted, in order to study and elaborate the fracture mechanics as well as to find any possible correlations with the mechanical property results.

## 4. Discussion

### 4.1. Mechanical Properties

Regarding the effect of continuous recycling processes on the mechanical properties of PA12, it was proved in this study that the mechanical properties overall increase until the 4th recycling course regarding the tensile strength, while there is an overall increase until the 3rd recycling course in flexural strength. Moreover, the impact strength results follow a similar trend as the overall increase ceases at the 3rd recycling course.

Regarding the tensile strength results, as stated above, there is a notable increase in the tensile strength values until the 5th recycling course, with a peak value of 39.1 MPa on the 4th recycling course. This result proves there is a total 17.7% improvement in the tensile strength, when compared to virgin PA12 material. The same trend is visible on the tensile modulus of elasticity results that clearly show a peak value of 176.6 MPa on the 4th recycling course. It has to be noted that the tensile modulus of elasticity experimentally obtained values and sample’s behavior could be more precisely attributed to (i) the “filamentous” type of printing in a layer-by-layer manner of the 3D printed bulk objects, as well as (ii) some plausible defects/voids between the sample’s layers, due to the nature of 3D printing, that could act as stress accumulation points. From these micro-cracks and while the material is being stressed at an external quasi static tensile field, it can be envisaged that some cracks could initiate and propagate in the longitudinal direction that the tensile test stress field is applied, resulting into a “plasticization”-like effect and yielding further to generally lower tensile modulus of elasticity values or, in other words, a knock-down effect on the material’s modulus. Specifically, similar behavior has been also observed for ABS, PLA, HDPE and PP as reported in our previous recent study [37], corroborating the behavior that was also experimentally determined for PA12 tensile modulus of elasticity values in the study at hand.

Finally, tensile stress versus strain graphs, indicate that specimens of the 4th cycle behave in a “stiffer” manner, when compared to the specimens of the rest of the cycles. There is no similar work in literature yet available to directly correlate the above findings. Similar studies regarding laser sintering with PA12 indicate that PA12 shows improved tensile strength after recycling [38]. It was also noted that in the Selective Laser Sintering (SLS) processes with PA materials, the thermal stress of the unused material is strong enough to cause aging [39].

Flexural properties show a similar behavior. Flexural strength (and flexural modulus of elasticity) starts to decrease after the 2nd recycling step and significantly decrease after the 4th repetition. More specifically, the peak value in the flexure strength is visible in the 2nd recycling course in this case with a value of 44.1 MPa. This indicates an increase in the flexural strength of 15.4%, when compared to the virgin material. Flexural modulus of elasticity follows the same trend with a peak value of 882.6 MPa in the 2nd recycling course. In a similar way, from Figure 5b, stress/strain curves’ slope, it is derived that the 2nd recycling course specimens shown a “stiffer” behavior.

Regarding the impact strength results, it was shown that there is an increase in the impact strength until the 4th recycling course. A peak value of 61.5 kJ/m^2^ is present at the 3rd recycling course and marks a 47.3% increase in the impact strength, when compared to the 1st recycling course [24].

Finally, regarding the micro-hardness Vickers results, it was shown that a peak of 28.5 HV value was present at the 4th recycling course, stating a slight improvement of 4%, when compared to the 1st recycling course. The overall micro-hardness findings indicate that there is not a clear improvement or decrease in the micro-hardness of the polyamide 12 specimens. This can be due to the layering structure of the FFF specimens. No literature is yet available for the micro-hardness on the recycled PA12.

**Figure 12:**
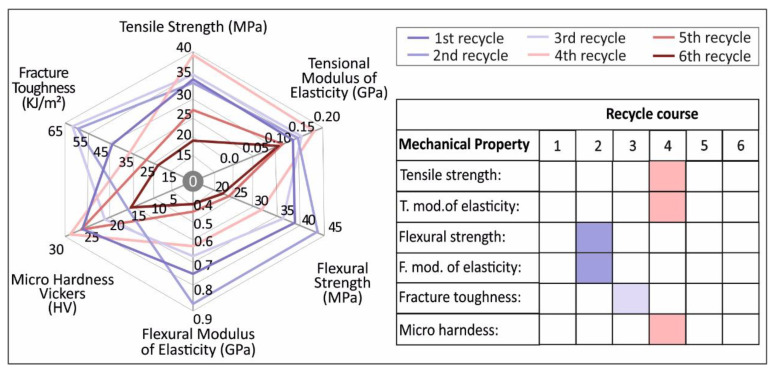
Virgin and recycled in the 6 recycle courses studied in this work PA12 mechanical properties summary.

Regarding the overall mechanical properties results (Figure 12), data from other studies [36,39,40,41] suggest that chain-scission and chain branching may occur simultaneously from the start of the reprocessing due to chain scission caused by the shear forces introduced in the extrusion system. These competing mechanisms alongside with a possible degree of crosslink might be the cause of the shift in the mechanical properties.

### 4.2. Thermal Analysis

Figure 8 depicts the overall thermal stability and critical degradation temperature of PA12 of 1st and 6th recycling courses, showing a slight degradation of the polymer. Moreover, the TGA graph shows the bandwidth and the maximum operating/processing temperature that the material can be processed by means of extrusion melting and 3D printing. TGA data indicates that a possible knock-down effect might be present due to the aforementioned degradation, affecting the thermal stability of the polymer and thus resulting in specimens with inferior mechanical properties after multiple recycling courses. This is in agreement with the tensile and flexure strength results of the Section 3.1 and Section 3.2 indicating that mechanical properties are indeed deteriorating after the 4th recycling course. Finally, the TGA curves show that the working/process temperature utilized in this work for all PA12 samples, both for recycling and 3D printing processes, are below the critical temperature of 401 °C, where the material in all cases starts rapidly to degrade with an abrupt weight loss.

DSC results in Figure 9 and Table 1 indicate a slight decrease in the degree of crystallinity with the recycling course, while there is not any noticeable shift in the corresponding melting (T_m_) and crystallization (T_c_) temperatures. The glass transition temperature (T_g_) for all samples was determined by the melting endotherm curves (Figure 9b), while it has been found in the temperature range of 50–55 °C. As it can be seen from Figure 9b, all samples exhibited similar crystallization and melting behavior, revealing thus that the crystallinity of the samples has not been significantly altered and/or affected by the multiple consecutive recycling and 3D printing processing cycles, until the 6th recycling course.

Literature suggests that decrease in crystallinity generally leads to the reduction of the effect that material molecules can pack and form crystal regions. The changes in crystallinity that are evident from the recycling procedure, more profound on the 6th recycling course, suggest that there might be a simultaneous occurrence of chain branching or polymer cross-linking along with chain scission, when using extrusion systems in the recycling process [36,42]. Furthermore, changes in the degree of crystallinity may result in changes of mechanical properties, i.e., creep, modulus, and hardness, presented in continuation. It should be mentioned that (i) some variation in the polymer chain lengths (chain scission) due to the consecutive recycling courses, affecting also a possible high quality of crystallite formation and the resulting polymer’s degree of crystallinity, accompanied with some plausible crosslinking of the polymer chains in the final 5th and 6th recycling courses are most likely the cause of the decrease in mechanical properties, as well as the inability to process further the material as it could no longer flow through the extrusion systems after the 6th recycling course.

Another interesting finding to mention from the DSC analysis shown with a dashed circular line in Figure 9b; specifically, in the melting endotherm curves for all samples, is an observed peak at ~155 °C. This is most likely attributed to cold recrystallization behavior of the samples, known to occur during the heating cycle and the endotherm curves before the melting endotherm peak. This phenomenon is explained by the fact that glassy fraction/part of the polymeric material (amorphous polymer chains) crystallizes with the thermal energy provided to the sample during the heating cycle. It was also evident that in the 6th recycling course there was found a slight shift of about +5 °C, while being slightly increased from the 1st to the 6th recycling course. The fact that the cold recrystallization is more prominent for the material with the increased recycled courses is explained by the fact that due to recycling/extrusion consecutive cycles, a higher fraction of short chains could be found. At the same time, above the T_g_ that the material/polymeric chains get some mobility, the polymeric chains that are in amorphous (glassy) state could more easily crystallize exhibiting thus a more intense cold recrystallization behavior for the 6th recycling course sample.

### 4.3. Morphological Characterization

As it can be seen from Figure 10, the specimens from recycling courses 1st to 4th exhibit uniform layer thickness and deposition, without any observed voids and/or discontinuities in the interlayers, most likely attributed to the optimum 3D printing parameters used in the current study, as well as the slight plausible degradation of the PA12 polymeric chains by the consecutive recycling cycles that can affect further the homogeneity of the polymer melt viscosity (affecting thus the 3D printing FFF process and the final quality of the 3D printed object).

The 5th and 6th recycling courses, on the other hand, show discontinuities in the layering and gaps from poor interfusion layering. These structural faults directly affect the mechanical properties of the 5th and the 6th recycling courses as the tensile/flexural and impact results showed.

Moreover, as it can be seen from the tensile specimens’ fracture area in Figure 11, specimens of the 6th recycling course had poor inter-layering fusion that led to the partial detachment/separation of the 2 outer (shell) layers of the printed parts; thus, tensile strength results for the 6th cycle indicated poor stress loading capacity.

Regarding the fractography of the samples, it was evident that the fracture mode changes after each recycling course. More specifically, it was shown that the failure mode of the specimens is initially more ductile, and throughout the recycling courses it becomes more brittle by each recycling step. Specimens from the 1st recycling course (Figure 11a) failed in a “ductile” manner than the samples of the 5th (Figure 10e) and 6th (Figure 10f) recycling courses. This brittle fracture mechanism observed by the SEM fractography for the 3D printed PA12 samples after the 3rd and 6th recycling courses (Figure 10c,f, respectively) is in agreement with the experimental stress–strain curves acquired in this work that demonstrate the stiffness and ductility of the different PA12 materials after each course of recycling. All the above could be more precisely attributed to the plausible chain scission and chain branching occurring phenomena during the material’s recycling and reprocessing steps, as have been well-documented by the corresponding DSC, TGA analyses in this study.

## 5. Conclusions

This research studies the effect of the thermomechanical treatment during the recycling process on the mechanical and thermal properties of PA12 polymer, over a certain number of recycling courses. In total, six (6) recycling courses were conducted, and tensile, flexural and impact properties along with micro-hardness were determined on FFF specimens. The following were derived:

1. The findings of this study prove that the overall mechanical behavior of the recycled PA12 polymer is generally improved over the recycling steps, for a certain number of repetitions, making PA12 a suitable polymer to be used in circular use.
2. It became evident that the recycling steps alter the mechanical properties of PA12 polymer, resulting in an average 15% increase in all mechanical properties studied herein, between the second and 3rd recycling course, while the polymer seems to be rapidly degrading after the fifth recycling course.
3. The crystallinity of PA12 polymer decreased slightly with the increase of the extrusion’s cycles, and the cross-linking and branching predominated over chain scission during multiple extrusions, thus the increase in the mechanical properties until the 3rd cycle (no reduction in crystallinity yet) and the decrease in the mechanical properties from 5th to 6th recycling course.
4. The materials’ flow was jeopardized after the 5th recycling course, making the filament hard to 3D print due to flow interruption. A possible low-grade crosslinking might be present affecting the flow rate of the recycled material.

## Figures and Tables

**Table 1:** Basic thermal characteristics of the PA12 samples as obtained and calculated from DSC results.

Sample	T_c_ (°C) Crystallization Temperature	T_m_ (°C) Melting Temperature	X_c_ (%) Crystallinity Degree
PA12 1st cycle	147	175	36.5
PA12 3rd cycle	144	174	36.1
PA12 6th cycle	145	175	30.6

## Data Availability

The data presented in this study are available on request from the corresponding author.
